# High Dietary Histamine Induces Digestive Tract Oxidative Damage in Juvenile Striped Catfish (*Pangasianodon hypophthalmus*)

**DOI:** 10.3390/antiox11112276

**Published:** 2022-11-17

**Authors:** Yu Liu, Xinlangji Fu, Huajing Huang, Jiongting Fan, Hang Zhou, Junming Deng, Beiping Tan

**Affiliations:** 1 College of Fisheries, Guangdong Ocean University, Zhanjiang 524088, China; 2 Aquatic Animals Precision Nutrition and High-Efficiency Feed Engineering Research Centre of Guangdong Province, Zhanjiang 524088, China; 3 Key Laboratory of Aquatic, Livestock and Poultry Feed Science and Technology in South China, Ministry of Agriculture, Zhanjiang 524088, China; 4 Laboratory of Aquatic Animal Nutrition and Feed, College of Fisheries, Guangdong Ocean University, Zhanjiang 524088, China

**Keywords:** histamine, striped catfish, digestive tract, mucosal barrier, oxidative damage

## Abstract

A 56-day feeding trial investigated the effects of dietary histamine on the antioxidant capacity, gastric and intestinal barrier functions, and growth performance of striped catfish (*Pangasianodon hypophthalmus*). Seven isonitrogenous (34.0% crude protein) and isolipidic (10.5% crude lipid) diets were formulated with supplemental 0, 15, 30, 60, 120, 240, and 480 mg/kg of histamine, named H0, H15, H30, H60, H120, H240, and H480 group, respectively. Results showed that the weight gain rate, specific growth rate, relative intestinal length in the H240 and H480 groups, and the condition factors in the H480 group were significantly lower than those in the H0 group. Intestinal total antioxidant capacity, peroxidase, catalase, superoxide dismutase, glutathione peroxidase, and glutathione reductase activities in the H480 group were significantly lower than those in the H0 group, whereas intestinal malondialdehyde content exhibited the opposite trend. Intestinal complement 3, complement 4, immunoglobulin M, and Recombinant Mucin 2 in the H480 group were significantly lower than those in the H0 group, in contrast to intestinal lipopolysaccharide content. Intestinal *IL-10* gene expression in the H480 group was significantly lower than that in the H0 group, whereas the *TNF-α*, *IL-1*, *IL-6*, and *IL-8* gene expression exhibited opposite results. Scanning and transmission electron microscopic observation of the gastrointestinal tract revealed severe damage to the gastric mucosa and intestinal epithelium in the H480 group. The abundance of *Treponema* in the histamine groups was significantly higher than that in the H0 group. These results indicated that high dietary histamine decreases intestinal immunity and antioxidant capacity, inducing digestive tract oxidative damage and ultimately decreasing the growth of striped catfish.

## 1. Introduction

The striped catfish (*Pangasianodon hypophthalmus*) is native to the Mekong and Chao Phraya River basins and is widely farmed in many Asian countries because of its fast growth, strong adaptability, and high disease resistance [1]. In Vietnam, exports of striped catfish already accounted for 23.2% of the national aquatic product exports in 2019 [2]. Due to its delicious meat quality, no intermuscular spines, and low price, striped fish is popular among consumers [3]. As a result, the culture scale of this species is still expanding. It is well known that reducing farming costs is one of the main strategies to improve farming economic efficiency [4]. In modern aquaculture, feed costs usually account for more than 50% of total costs, since fish meal is increasingly expensive [5,6,7]. Currently, fish meal remains an integral part of commercial feed for striped catfish, remaining at 20–60% [8]. In Vietnam, stale fishmeal has been routinely used in catfish farming because of its local availability and it facilitates the control of feed costs [9]. However, fishmeal stored for long periods usually contains high doses of histamine due to the decarboxylation of histidine [10,11,12]. Moreover, striped catfish are cultivated commercially in subtropical and tropical regions where unfavorable storage conditions (humidity and heat) often lead to feed spoilage and produce large amounts of histamine [13]. Therefore, striped catfish may face dietary challenges with high doses of histamine.

Histamine is a low molecular weight organic nitrogen compound with the chemical formula of C5H9N3 [14], which extensively participated in numerous metabolic activities [15,16,17]. Excessive intake of histamine may cause human health disorders, poisoning, headaches, and even anaphylaxis [18,19,20]. Therefore, some regions and countries legislate to limit the histamine content in food; for the European Union, Australia, and South Africa, histamine content should be less than 100 mg/kg [21,22,23], and for the United States of America, below 50 mg/kg [24]. Recently, the effects of histamine on the growth, metabolism, and health of aquatic animals have attracted widespread attention [12,25,26,27,28,29], with results showing that dietary histamine interferes with normal metabolism, causes inflammatory responses, and reduces antioxidant capacity and growth in aquatic animals. Moreover, the toxic effects of dietary histamine can directly damage the morphology, physiology, and health of the digestive tract of aquatic animals [29,30,31,32] and disrupt the normal gut microbiota structure [28]. Thus, striped fish may suffer from the toxic effects of dietary histamine; however, limited information is available about this.

The digestive tract is the main nutrient intake place of fish, and its physiological function and health maintenance are closely related to antioxidant and immune status. Specifically, the antioxidant status usually depends on the activity of antioxidant enzyme systems, including peroxidase, catalase superoxide dismutase, glutathione peroxidase, and glutathione reductase; the immune status is determined by both the innate and acquired immune systems, with the innate immune system being the dominant type in fish [29], which consists of complement and immunoglobulins. To date, no studies have reported the effects of dietary histamine on the intestinal antioxidant and immune system of striped catfish. Therefore, this study evaluated the effects of dietary histamine on the antioxidant capacity, gastric and intestinal barrier functions, and growth performance of striped catfish. Our data will contribute to the healthy farming of striped fish.

## 2. Materials and Methods

### 2.1. Experimental Diets Preparation

Before making the test diet, we tested the histamine content of 22 commercial feeds for striped catfish (Table 1), and the results showed that the histamine contents ranged from 33.70–177.00 mg/kg. Among them, the histamine content exceeded 80 mg/kg in eight feeds, accounting for 36.36%. Subsequently, the histamine content of the experimental diet was designed based on these data.

Seven isonitrogenous (34.0% crude protein) and isolipidic (10.5% crude lipid) diets were formulated with supplemental 0, 15, 30, 60, 120, 240, and 480 mg/kg of histamine, named H0, H15, H30, H60, H120, H240, and H480 groups, respectively. To reduce the histamine content in the basal diet, we used fresh white fish meal (PILENGA 2, histamine ≤ 40 mg/kg, purchased from fishing vessel IMO: 9120310). All ingredients were ground into meals and sifted out with a 60 μm sieve, and then accurately weighed and mixed with a V-mixer (M-256, South China University of Technology, Guangzhou, China). Subsequently, the soybean oil and pure water were supplemented to prepare a dough and then extruded with an extruder (School of Chemical Engineering, South China University of Technology, Guangzhou, China) to produce 2.0 mm diameter pellets. The moist pellets were dried in an air-conditioned room at 25 °C (dehumidification mode) and then collected in self-sealing bags and stored at −20 °C until use. Diet formulation and proximate composition are shown in Table 2.

### 2.2. Animal and Feeding Trial

A total of 630 striped catfish juveniles (body weight = 31.38 ± 0.09 g) were randomly assigned to 21 cages with 30 fish per cage (0.7 m × 0.7 m × 1 m). The feeding trial lasted 8 weeks, and the fish were fed twice daily at 7:00 and 17:30 h to apparent satiation during this period. The amount of feed fed to each net box was recorded daily and checked for fish mortality. This trial was conducted in the Freshwater Base of Guangdong Ocean University with the following water parameters: temperature keep at 29–32 °C, dissolved oxygen > 4.0 mg/L, ammonia nitrogen < 0.04 mg/L.

### 2.3. Sample Collection

At the end of the feeding trial, the fish were starved for 1 day, then weighed and counted. Subsequently, the fish were anesthetized with eugenol solution (1:10,000 dilution, Macklin, Shanghai, China) before starting the sampling. Four fish per cage were individually measured in terms of body weight and length, visceral weight, liver weight, intestinal length, and intestinal weight to calculate condition factors (CF), viscerosomatic index (VSI), hepatosomatic index (HSI), relative intestinal length (RIL) and relative intestinal weight (RIW). Four fish from each cage were selected for dissection, and the hindgut tissues were harvested and placed in an Eppendorf (EP) tube, then stored at −80 °C for enzyme activities analysis. Another four fish per cage were selected for dissection, and the hindgut was collected in an RNAlater-added EP tube for intestinal gene quantitative analysis, and the digesta in the hindgut was collected for intestinal flora analysis.

### 2.4. Gastrointestinal Tract Histomorphological Observation

One fish per cage from the H0, H60, and H480 groups was selected for dissection, and the stomach and hindgut were harvested and placed in an EP tube containing glutaraldehyde fixative (Wuhan Servicebio Technology Co., Ltd., Wuhan, China). Subsequently, the stomach tissue blocks were washed with 0.1 mol/L phosphoric acid buffer (PSB) solution 3 times, 15 min each, and then the tissue was transferred to PBS containing 1% OsO4 and left for 1–2 h at room temperature. Afterward, the tissue blocks were washed three times with PBS for 15 min each, followed by dehydration with an alcohol gradient. The tissues were placed in an isoamyl acetate solution and then dried with a critical point dryer (K850, Quorum, Nottingham, United Kingdom). Finally, the specimens were attached to metal stakes for 30 s using a carbon sticker and sputtering apparatus (MC1000, Hitachi, Tokyo, Japan) and then observed using a scanning electron microscope (SEM, SU8100, Hitachi, Japan).

Hindgut tissue was fixed and dehydrated according to the procedure of gastric tissue, and the treated hindgut tissue was placed in resin for permeabilization and embedding, followed by cutting into ultrathin sections (Leica UC7, Leica, Wetzlar, Germany). Subsequently, ultrathin sections were stained with 2% uranyl acetate and 2.6% lead citrate and observed using a transmission electron microscope (TEM, HT7800, HITACHI, Japan).

### 2.5. Intestinal Biochemical Parameters Analysis

Wet intestine samples were first accurately weighted, and then a ninefold volume (v/m) of phosphate buffer (pH 7.4) was added to prepare crude enzyme extract solution with the following method: homogenized evenly using a homogenizer (IKA Works Asia, Bhd, Kuala Lumpur, Malaysia) and then centrifuged at 875 g for 10 min at 4 °C; the supernatant was then removed for subsequent analysis. The total protein concentration of each intestine sample was measured by the bicinchoninic acid method (BCA) using the commercially available kit (No. A045-3-2, Nanjing Jiancheng Bioengineering Institute, Nanjing, China). Intestinal total antioxidant capacity (T-AOC, ABTS method, No. A015-2-1), peroxidase (POD, colorimetric method, No. A084-1-1), catalase (CAT, ultraviolet method, No. A007-2-1), superoxide dismutase (SOD, WST-1 method, No. A001-3-2), glutathione peroxidase (GPX, colorimetric method, No. A005-1-2), glutathione reductase (GR, ultraviolet method, No. A062-1-1), and lysozyme (LZM, turbidimetric method, A050-1-1) activities and intestinal malondialdehyde (MDA, TBA method, No. A003-1-2) content were determined by using commercial kits purchased from Nanjing Jiancheng Bioengineering Institute (Nanjing, China). Intestinal recombinant lipopolysaccharide (LPS, No. ml505648), complement 3 (C3, ml003460), complement 4 (C4, ml003461), and immunoglobulin M (IgM, No. ml326413) contents were determined by the ELISA method using commercial kits purchased from Shanghai Enzyme Link Biotechnology Co., Ltd. (Shanghai, China). The determination procedures of each parameter were performed in strict accordance with the corresponding instructions, and unit conversion was performed by dividing by the protein concentration of the corresponding sample.

### 2.6. Real-Time Quantitative PCR

Total intestinal RNA was extracted using the TRIzol™ Reagent kit (TransGenBiotech, Beijing, China), followed by concentration and integrity detection using a spectrophotometer (NanoDrop^®^ ND-2000, Thermo, Waltham, MA, USA) and a 1.2% denatured agarose, respectively. Subsequently, the qualified samples (1 μg RNA) were used as reverse transcription templates to synthesize cDNA using commercial kits (Accurate Biology, Changsha, China). Real-Time Quantitative PCR analysis was performed under a 10 μL SYBR^®^ Green Premix Pro Taq HS qPCR Kit II reaction system (Accurate Biology, China) using a quantitative PCR instrument (Roche LightCycler^®^ 480) following the method described in a previous study [33]. Briefly, the 10 μL reaction system contains a 10 ng cDNA template, 0.4 μM forward primers, 0.4 μM reverse primers, 5 μL 2 × SYBR^®^ Green Pro Taq HS Premix II, and the rest is RNase-free water. The PCR instrument was set up with a program of denaturation at 95 °C for 30 s, followed by 40 amplification cycles with denaturation at 95 °C for 5 s and annealing at 60 °C for 30 s. The primers used in this trial are shown in Table 3. The expression level or target genes were calculated by the 2^−ΔΔCT^ method [34] and normalized with the expression level of β-actin in the H0 group.

### 2.7. Intestinal Microbiome Analysis

Intestinal microbiota total DNA was extracted by using the HiPure Soil DNA Extraction Kit (Magen, Guangzhou, China) according to the instructions, followed by a quality test with a UV spectrophotometer (Thermo, Waltham, MA, USA). Subsequently, the V3-V4 region of the 16SrRNA gene was amplified using a universal primer pair (341F/806R, CCTACGGGNGGCWGCAG/GGACTACHVGGGTATCTAAT) and a commercially available kit (New England Biolabs, MA, USA). The amplification conditions were set as follows: pre-denaturation at 95 °C for 5 min, followed by denaturation at 95 °C for 1 min, annealing at 60 °C for 1 min, extension at 72 °C for 1 min, and incubation at 72 °C for 7 min after performing 30 cycles. PCR amplification was performed using a 50 μL reaction system containing 10 μL of 5 × Q5@ Reaction Buffer, 10 μL 5 × Q5@ High GC Enhancer, 1.5 μL of 2.5 mM dNTPs, 1.5 μL of forward and reverse primers (10 μM), 0.2 μL of Q5@ High-Fidelity DNA Polymerase, and 50 ng of template DNA. Afterward, the amplification products are purified and quantified, and then a library is constructed before initiating the sequencing work (Illumina platform, Illumina, San Diego, CA, USA). Intestinal microbiome analysis was entrusted to Guangzhou Genedenovo Biotechnology Co., Ltd. (Guangzhou, China).

### 2.8. Statistical Analysis

The obtained data were subjected to one-way analysis of variance (ANOVA) by SPSS software (version 22, IBM, Chicago, IL, USA), followed by Duncan’s multiple comparison test when *p*-value < 0.05. All data were expressed as mean ± standard error of measurement (SEM).

## 3. Results

### 3.1. Growth Performance and Morphologic Indexes

As shown in Table 4, the SR, VSI, and RIW were not significantly affected by experimental diets (*p* > 0.05). The FMW, WGR, SGR, and RIL in the H240 and H480 groups and the CF in the H480 group were significantly lower than those in the H0 group, whereas the FCR in the H240 and H480 groups exhibited an opposite result (*p* < 0.05). The HIS in the H0 group was significantly lower than that in the H60, H120, H240, and H480 groups (*p* < 0.05).

$$WGR\left(\%\right)=100\times (W_{F}-W_{I})/W_{I}$$

$$SGR\left(\%/\mathrm{day}\right)=100\times \left(\mathrm{Ln}W_{F}-\mathrm{Ln}W_{I}\right)/56$$

$$FCR\left(\%\right)=100\times W_{\mathrm{FI}}/\left(W_{F}-W_{I}\right)$$

$$SR\left(\%\right)=100\times N_{F}/N_{I}$$

$$VSI\left(\%\right)=100\times W_{V}/W$$

$$HSI\left(\%\right)=100\times W_{L}/W$$

$$CF\left(g/cm^{3}\right)=W/L^{3}$$

$$RIL\left(\%\right)=100\times L_{I}/L$$

$$RIW\left(\%\right)=100\times W_{I}/W$$

where *W*_I_, *W*_F_, *W*_FI_, *N*_I_, and *N*_F_ are the initial body weight (g), final body weight (g), feed intake weight (g), initial fish numbers, and final fish numbers, respectively; *W*, *W*_V_, *W*_L_, *W*_I_, *L*, and *L*_I_ are the body weight (g), visceral weight (g), liver weight (g), intestinal weight (g), body length (cm), and intestinal length (cm) of the same sampled fish. 

### 3.2. Intestinal Antioxidant Capacity

As shown in Table 5, intestinal POD and SOD activities in the H480 groups, intestinal GR activity in the H240 and H480 groups, and intestinal T-AOC and GPX activities in the H120, H240, and H480 groups were significantly lower than those in the H0 group, whereas the intestinal MDA content in the H480 group exhibited an opposite result (*p* < 0.05). The intestinal CAT activity in the H30, H60, H120, H240, and H480 groups was significantly lower than that in the H0 group (*p* < 0.05).

### 3.3. Intestinal Immune Status

As shown in Table 6, the intestinal LZM activity was not significantly affected by experimental diets (*p* > 0.05). The intestinal C3 content in the H240 and H480 groups and the IgM content in the H60, H120, H240, and H480 groups were significantly lower than those in the H0 group (*p* < 0.05). The intestinal C4 content in the H30, H60, H120, H240, and H480 groups was significantly lower than that in the H0 group, whereas the LPS content exhibited an opposite result (*p* < 0.05).

### 3.4. Intestinal Inflammatory Response

**Figure 1:**
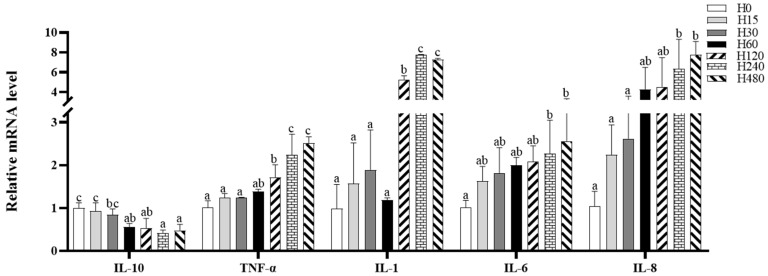
Intestinal inflammation-related gene expression of striped catfish fed with experimental diets. Values in each column with different superscripts represent significant differences (*p* < 0.05).

The intestinal IL-6 and IL-8 expressions in the H240 and H480 groups and the intestinal TNF-α and IL-1 expressions in the H120, H240, and H480 groups were significantly higher than those in the H0 group, whereas the IL-10 expression in the H60, H120, H240, and H480 groups was significantly lower than that in the H0 group (*p* < 0.05; Figure 1).

### 3.5. Gastric SEM and Intestinal TEM Observations

**Figure 2:**
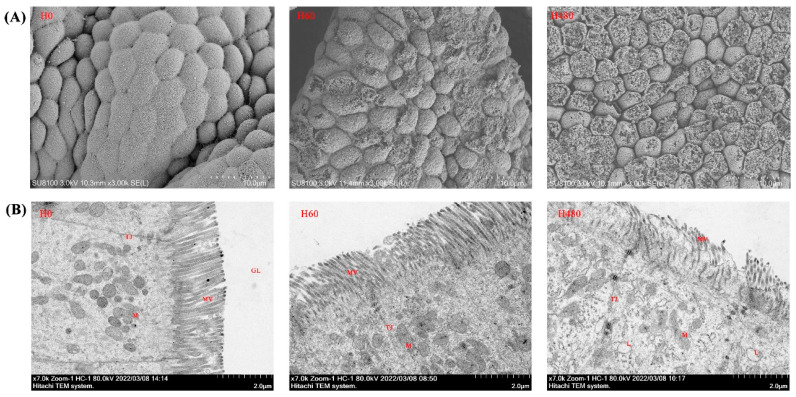
Gastric SEM and intestinal TEM observations of striped catfish fed with experimental diets. (**A**), gastric SEM observations; (**B**), intestinal TEM observations. M, mitochondria; TJ, tight junction; MV, microvillus; L, lysosome. Mitochondrial dissolution, lysosome volume increase, and microvilli damage were clearly observed in the H480 group.

The gastric SEM and intestinal TEM observations are presented in Figure 2A,B, respectively. As shown in Figure 2A, the degree of gastric mucosal damage increased with increasing dietary histamine levels. Furthermore, as shown in Figure 2B, dietary histamine damaged intestinal epithelial cells, and organelle lysis was observed in the H480 group.

### 3.6. Microbiota Structure

**Figure 3:**
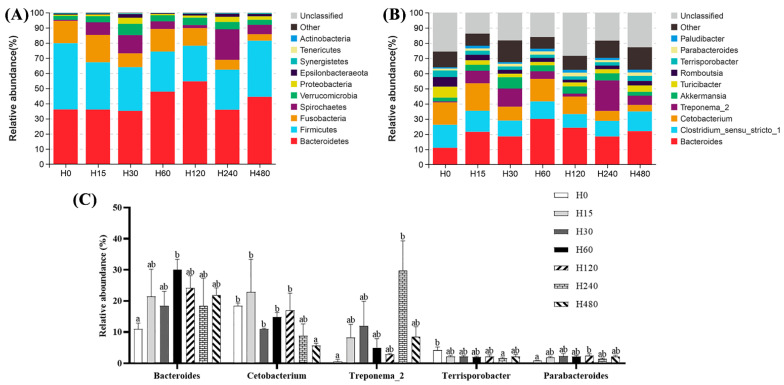
Intestinal flora composition of striped catfish fed with different experimental diets. (**A**), phylum level; (**B**), general level; (**C**), genera with significant differences. Values in each column with different superscripts represent significant differences (*p* < 0.05).

As shown in Table 7, the Goods coverage of all the groups was above 99%, indicating that the sequencing depth in this study was adequate. The Shannon, Simpson, Chao1, and ACE indices were not significantly affected by the experimental diets (*p* > 0.05). The structural composition analysis of microbiota exhibited that Bacteroidetes, Firmicutes, Fusobacteria, and Spirochaetes were the dominant phyla of all groups (Figure 3A). The top 10 genera of all groups are shown in Figure 3B, and the *Bacteroides*, *Clostridium sensu stricto*, and *Cetobacterium* were the three dominant genera in all groups. As shown in Figure 3C, the abundance of *Bacteroides* in the H60 group, the abundance of Treponema2 in the H240 group, and the abundance of *Parabacteroides* in the H120 group were significantly higher than those in the H0 group, whereas the abundance of *Catobacterium* in the H480 group and the abundance of *Terrisporobacter* in the H240 group were significantly lower than those in the H0 group (*p* < 0.05).

## 4. Discussion

This study first investigated the effects of dietary histamine on the antioxidant capacity, gastric and intestinal barrier functions, and growth performance of striped catfish. As a toxic dietary component, histamine has attracted much attention from aquatic animal nutritionists in recent years. Previous studies have shown that dietary histamine significantly decreased the growth performance of the juvenile group (*Epinephelus coioides*) [35], mysis (*Neomysis japonica Nakazawa*) [36,37], American eels (*Anguilla rostrata*) [38,39], yellow catfish (*Pelteobagrus fulvidraco*) [29], and rainbow trout (*Oncorhynchus mykiss*) [40]. In this study, our data exhibited that dietary histamine below 60 mg/kg has limited effects on the growth of striped catfish, whereas dietary histamine above 120 mg/kg significantly decreased the growth, suggesting that the growth-inhibiting effect of dietary histamine was dose-dependent. Of note, the striped catfish in the H15 group showed better growth than in the H0 group. Similarly, He et al. (2018) [40] found that diets containing 18.0 mg/kg of histamine had a growth-promoting effect on yellow catfish. This evidence suggests that low-dose histamine may benefit the growth of fish. Conversely, diets containing 4000 mg/kg of histamine showed no detrimental effects on the performance of Chinese mitten crab (*Eriocheir sinensis*) [32], and high-dose dietary histamine (2400 mg/kg) even showed a growth-promoting effect on blue shrimp (*Litopenaeus stylirostris*) [41]. The difference in these results may be related to differences in the digestive physiology of different aquatic animals.

A healthy digestive tract is important for maintaining proper immunity and growth in fish, and the health of the digestive tract is regulated by dietary ingredients [42,43]. Oxidative damage is an unavoidable part of the physiological activity of aerobic organisms, due to the continuous production of oxygen radicals during their physiological metabolism [44]. The scavenging of oxygen radicals is accomplished by the antioxidant system, including antioxidant enzymes and antioxidant active substances [45]. Therefore, the antioxidant capacity is commonly used to evaluate the health status of fish. In this study, fish in the H480 group exhibited the lowest intestinal T-AOC, POD, CAT, SOD, GPX, and GR activities and the highest MDA content, suggesting that high dietary histamine is detrimental to the intestinal antioxidant capacity of striped catfish. Similarly, high-dose dietary histamine significantly decreased the antioxidant capability of grouper [35], American eel [38], and Pacific white shrimp (*Litopenaeus vannamei*) [12]. Liu et al. [35] concluded that the long-term intake of histamine disrupts the ability of fish to respond to reactive oxygen intermediates, thereby disrupting the antioxidant system and causing oxidative damage. However, considering that the intestinal antioxidant capacity of striped catfish fed low-dose dietary histamine (below 60 mg/kg) was not significantly reduced, we hypothesized that dietary histamine disrupts the intestinal antioxidant system in a dose-dependent manner.

Intestinal innate immunity plays an essential role in maintaining intestinal health, which constitutes the first line of defense against colonization by disease-causing microorganisms [46]. Intestinal immune activity is accomplished by the synergistic action of multiple immune enzymes and immunologically active substances, including immunoglobulins M (IgM), lysozyme, and the complement (C3, C4) [47,48]. In this study, dietary histamine (above 120 mg/kg) significantly decreased intestinal C3, C4, and IgM contents, suggesting that high-dose dietary histamine is detrimental to the innate immunity of striped catfish, thereby disrupting intestinal health. Besides, the complement system also plays an equally important role in regulating inflammation in fish [49]. The inflammatory response is usually regulated by a dynamic balance of pro- and anti-inflammatory factors [26,50]. In this study, dietary histamine (above 120 mg/kg) caused the downregulation of anti-inflammatory factors (e.g., IL-10 and NF-κB) and the upregulation of pro-inflammatory factors (e.g., IL-1, IL-6, IL-8, and TNF-α), suggesting that high doses of histamine may cause intestinal inflammation in striped catfish. Similarly, dietary histamine also induced inflammation in Pacific white shrimp and Perciformes [12,51]. Galindo-Villegas et al. [51] suggested that dietary histamine regulates the inflammatory response by acting directly with macrophages. Therefore, this evidence suggests that high doses of dietary histamine may trigger an inflammatory response mediated by macrophages, thereby impairing intestinal health.

Dong et al. [52] suggested that inflammation usually occurs when immune cells are infected or tissues are damaged. In this study, the intestinal TEM observation exhibited that high-dose dietary histamine (480 mg/kg) severely damaged the structure and morphology of intestinal epithelial cells, including an increase in the number of lysosomes and swelling of mitochondria and endoplasmic reticulum. Moreover, the gastric SEM observation also showed that high-dose dietary histamine severely damaged the mucosal layer. Combined with the poor intestinal antioxidant capacity and upregulated inflammation levels, these results sufficiently suggest that high-dose dietary histamine disrupts the intestinal antioxidant system, causing oxidative damage to intestinal tissues and inducing inflammation in striped catfish. More importantly, the morphology of the digestive tract is closely related to its physiological function [53]. Thus, the poor digestive tract morphology also plausibly explains the reduced intestinal immune function and growth performance of striped catfish [54]. Similar results were observed in grouper [35] and yellow catfish [29]. This evidence suggests that high-dose dietary histamine may reduce fish growth by disrupting digestive tract health and function.

As a component of the intestinal mucosal layer, the intestinal flora is also critical to intestinal health and function [55,56]. Meanwhile, the structure of the intestinal flora is easily affected by dietary ingredients [57,58]. In this study, Firmicutes, Fusobacteria, Bacteroidetes, and Spirochaetes were the four dominant phyla of all groups, which was highly consistent with a previous study reported by Hieu et al. [59]. These results indicate that these phyla may constitute the core flora of striped catfish and are essential for maintaining intestinal health and function [60]. In this study, dietary histamine altered the relative abundance of individual phyla, although it did not change the species of the core flora, suggesting that dietary histamine may induce the migration of intestinal function.

In this study, Fusobacteria, mainly *Cetobacterium* genera, have been reported to produce vitamin B_12_, acetate, and propionate through their fermentation process [61,62]. These metabolites extensively participate in the regulation of energy metabolism, gut health, and gut microecology [62,63]. Therefore, a decrease in the abundance of *Cetobacterium* in the H480 group suggests that high-dose dietary histamine is detrimental to the intestinal physiological function and health of striped catfish. *Bacteroidetes* mainly consist of Bacteroides and Parabacteroides in this trial. *Bacteroidetes* are considered to be the main catabolite of polysaccharides in the intestine, producing large amounts of short-chain fatty acids that contribute to improved intestinal health [64]. *Parabacteroides* are a butyrate producer with equally positive effects on improving intestinal health [65,66]. Thus, an increase in the abundance of *Bacteroides* and *Parabacteroides* may suggest that low-dose dietary histamine has an ameliorative effect on intestinal health. Nevertheless, this degree of ameliorative effect did not effectively mitigate the toxic effects of dietary histamine, and therefore, dietary histamine ultimately impaired the intestinal health of striped catfish. Moreover, considering that dietary histamine caused a significant decrease in intestinal maltase activity in striped catfish (Table S1) and that both *Bacteroides* and *Parabacteroides* usually use carbohydrates as the carbon source, we speculate that the increase in the abundance of these two genera caused by dietary histamine may be related to poor carbohydrate utilization efficiency. Firmicutes mainly consist of *Terrisporobacter* genera in this study. *Terrisporobacter* is an acetate-producing bacterium that is positively correlated with host health [67,68]. *Treponema_2* genera are the dominant genera of Spirochaetes in this study, which has been reported as pathogenic bacteria [69]. Therefore, a decrease in the abundance of *Terrisporobacter* and a decrease in the abundance of *Treponema_2* in the H240 group suggests that high-dose dietary histamine is detrimental to the intestinal health of striped catfish. Moreover, the colonization of pathogenic microorganisms indicates a decrease in the immune viability of the intestinal mucosa and an imbalance in the balance of the microbial community. Combined with the poor intestinal immunity, decreased intestinal antioxidant capacity, and increased oxidative stress in the H240 group, the increased abundance of pathogenic microorganisms in the H240 group suggests that a high dose of dietary histamine disrupts intestinal antioxidant and immune defenses, leading to colonization by pathogenic microorganisms.

## 5. Conclusions

In conclusion, high-dose dietary histamine decreased intestinal antioxidant capacity, thereby inducing intestinal oxidative damage and decreasing the growth of striped catfish. In addition, high-dose dietary histamine reduces intestinal immunity, induces the colonization of pathogenic microorganisms and intestinal inflammation, and impairs intestinal health. High-dose dietary histamine severely damaged the mucosal layer of the digestive tract in striped catfish. More importantly, our data confirmed that histamine is a toxic dietary component for aquatic animals and that increases in histamine levels should be prevented during aquafeed production and storage.

## Figures and Tables

**Table 1:** Histamine content of commercial feeds.

Manufacturer	Product Name	Production Date	Test Date	Crude Protein (%)	Crude Lipid (%)	Histamine (mg/kg)
CPP	783-③	2021/3/25	2021/8/5	26.60	6.98	58.60
CPP	781-②	2021/4/16	2022/2/7	32.56	8.02	89.00
CPP	781	2021/7/9	2022/2/7	33.72	7.60	40.00
Evergreen	102-3	2021/11/21	2022/2/7	32.80	8.20	107.00
Evergreen	102-3	2021/6/24	2022/2/7	32.56	7.09	40.90
Foshan Baiyang	BC-3	2020/11/30	2021/5/27	29.08	6.37	53.80
GLOBAL	SAFIR-3	2021/6/11	2022/2/7	31.29	8.34	44.60
Indonesia Evergreen	104-3	2021/3/29	2021/8/5	23.04	8.72	50.00
Indonesia Evergreen	104-2	2021/6/19	2021/8/5	23.89	8.69	60.90
Indonesia Tongwei	168	2021/2/6	2021/8/5	32.03	7.92	67.80
Jiangmen Coral	3662	2021/1/25	2021/5/10	34.10	8.34	177.00
PT. CJ	AT-3	2021/3/28	2021/8/5	15.80	7.12	55.80
PT. SURITANI PEMUKA	SPM 4A	2021/4/5	2021/8/5	25.92	5.98	51.00
STP	LA 7K	2021/8/1	2022/2/7	29.69	7.21	33.70
Tongwei	8505	2021/7/18	2022/2/7	34.44	9.54	121.00
Yangjiang Dahai	3664	2020/9/27	2021/5/10	29.39	5.66	54.20
Yangjiang Dahai	3663	2021/8/2	2021/8/16	28.92	5.58	54.50
Yangjiang Dahai	3664	2021/9/3	2021/11/12	29.20	5.33	99.50
Yangjiang Dahai	3664	2021/9/4	2021/11/12	28.32	5.32	112.00
Yangjiang Dahai	4411	2021/10/10	2021/11/25	30.46	5.88	104.00
Zhanjiang Yuehua	3663	2021/5/9	2021/5/27	32.44	5.30	56.40
Zhanjiang Yuehua	3663	2021/5/29	2021/7/23	34.38	6.28	81.50

**Table 2:** Ingredients and nutritional compositions of the experimental diets.

Ingredients	H0	H15	H30	H60	H120	H240	H480
White fish meal	150.00	150.00	150.00	150.00	150.00	150.00	150.00
Rapeseed meal	200.00	200.00	200.00	200.00	200.00	200.00	200.00
Soybean meal	200.00	200.00	200.00	200.00	200.00	200.00	200.00
Wheat flour	150.00	150.00	150.00	150.00	150.00	150.00	150.00
Rice bran meal	252.99	252.99	252.99	252.99	252.99	252.99	252.99
Soybean oil	20.00	20.00	20.00	20.00	20.00	20.00	20.00
Ca(H_2_PO_4_)_2_	12.00	12.00	12.00	12.00	12.00	12.00	12.00
Choline chloride (50%)	4.00	4.00	4.00	4.00	4.00	4.00	4.00
Vitamin C	0.20	0.20	0.20	0.20	0.20	0.20	0.20
Compound premix ^a^	10.00	10.00	10.00	10.00	10.00	10.00	10.00
Histamine dihydrochloride	0.00	0.025	0.050	0.101	0.203	0.406	0.811
Cellulose microcrystalline	0.81	0.785	0.760	0.709	0.607	0.404	0.00
Proximate composition ^b^ (dry matter, g/kg)							
Moisture	104.76	109.79	105.13	104.49	105.47	97.18	105.76
Crude protein	338.00	342.94	342.44	338.50	347.50	353.81	348.88
Crude lipid	106.52	104.61	103.88	109.84	107.33	105.23	104.36
Ash	104.48	107.76	101.83	103.91	98.99	96.99	101.63
Gross energy (MJ/kg) ^c^	18.33	18.18	18.34	18.42	18.50	18.67	18.39
Histamine (mg/kg)	8.47	23.60	38.59	68.49	128.71	248.87	488.75

^a^ Compound premix (g/kg mixture): vitamin A, 0.20 g; vitamin D3, 0.003 g; vitamin E, 4.40 g; vitamin K3, 0.66 g; vitamin B1, 0.33 g; vitamin B2, 0.88 g; vitamin B6, 0.73 g; vitamin B12, 0.001 g; nicotinic acid, 2.89 g; calcium pantothenate, 1.64 g; folic acid, 0.07 g; biotin, 0.003 g; vitamin C, 10.01 g; FeSO_4_·7H_2_O, 52.87 g; H_3_ClCu_2_O_3_, 0.65 g; ZnSO_4_·7H_2_O, 43.15 g; MnSO_4_·7H_2_O, 31.56 g; MgSO_4_·H_2_O, 44.65 g; Ca(IO_3_)_2_, 0.42 g; Na_2_SeO_3_, 0.11 g; CoCl_2_·6H_2_O, 0.14 g. ^b^ Measured values; GE, gross energy; CP, crude protein; CL, crude lipid; DM, dry matter; ASH, ash. ^c^ Gross energy.

**Table 3:** Primers pair sequences used for real-time PCR.

Target Gene	Primer Sequence	Product Size	Accession No.
TNF-α	F-TGTCTCGCTGGTCTGACTCCTATG R-CAGTGGGTTTGTTGCTCTTCAAGTG	97	XM_026942329.2
IL-10	F-TCTACTTGGAGACCGTGTTGCCTAG R-GATGGTGTCGATGGGAGTTCTGAAG	80	XM_026935649.2
IL-6	F-GACTGCGGGTCTGAGAGTTTACTTC R-GCAACACTGGGTCTGATCTGTCTG	142	XM_026922014.2
IL-8	F-GCTTAGGGAGGTGAGGGCTGAG R-TAGGTGTGGAGGTGGATGTGGTAAG	112	XM_027138229.2
IL-1β	F-TTCTTCAGAAACGGCACTGGTGAC R-GGAGGTGACTGGATTGCTGCTTAC	130	NM_001200219
β-actin	F-GGCTACTCCTTCACCACCACA R-ATTGAGTCGGCGTGAAGTGGTAAC	100	XM_026929614.2

TNF-α, tumor necrosis factor a; IL-10, interleukin-10; IL-6, interleukin-6; IL-8, interleukin-8; IL-1β, interleukin 1-beta.

**Table 4:** Effect of dietary histamine on growth performance and morphologic indexes of striped catfish.

Items	H0	H15	H30	H60	H120	H240	H480
IBW (g)	31.45 ± 0.01	31.40 ± 0.08	31.35 ± 0.03	31.32 ± 0.02	31.33 ± 0.04	31.36 ± 0.14	31.43 ± 0.12
FBW (g)	106.56 ± 1.80 ^b^	107.68 ± 2.86 ^b^	105.25 ± 1.60 ^b^	105.00 ± 5.67 ^b^	102.87 ± 2.21 ^ab^	96.8 ± 5.69 ^a^	96.31 ± 2.90 ^a^
WGR (%)	2.39 ± 0.06 ^c^	2.43 ± 0.08 ^c^	2.36 ± 0.05 ^c^	2.31 ± 0.12 ^bc^	2.28 ± 0.08 ^abc^	2.09 ± 0.19 ^ab^	2.07 ± 0.08 ^a^
FCR (%)	1.27 ± 0.05 ^a^	1.26 ± 0.04 ^a^	1.30 ± 0.03 ^a^	1.33 ± 0.02 ^ab^	1.34 ± 0.04 ^ab^	1.48 ± 0.12 ^bc^	1.43 ± 0.06 ^c^
SGR (%/day)	2.18 ± 0.05 ^b^	2.20 ± 0.04 ^b^	2.16 ± 0.03 ^b^	2.14 ± 0.02 ^b^	2.12 ± 0.04 ^b^	1.99 ± 0.11 ^a^	2.00 ± 0.04 ^a^
SR (%)	100	100	100	100	100	100	100
HSI (%)	1.63 ± 0.08 ^a^	1.73 ± 0.20 ^ab^	1.83 ± 0.16 ^ab^	1.90 ± 0.19 ^b^	1.91 ± 0.18 ^b^	1.89 ± 0.18 ^b^	1.96 ± 0.23 ^b^
VSI (%)	11.19 ± 1.44	11.35 ± 1.94	11.03 ± 1.04	10.97 ± 1.67	11.41 ± 1.78	10.56 ± 1.79	10.74 ± 1.72
CF (g/cm^3^)	1.54 ± 0.05 ^b^	1.52 ± 0.04 ^b^	1.52 ± 0.06 ^b^	1.51 ± 0.06 ^ab^	1.50 ± 0.08 ^ab^	1.46 ± 0.10 ^ab^	1.43 ± 0.05 ^a^
RIL (%)	213.43 ± 28.21 ^b^	197.41 ± 9.7 ^ab^	195.42 ± 12.97 ^ab^	189.1 ± 19.91 ^ab^	183.31 ± 40.77 ^ab^	172.54 ± 29.43 ^a^	165.81 ± 29.95 ^a^
RIW (%)	1.71 ± 0.34	1.75 ± 0.25	1.91 ± 0.31	1.88 ± 0.31	1.74 ± 0.32	1.66 ± 0.28	1.64 ± 0.28

Data in the same row with different superscript letters indicate significant differences between groups (*n* = 3; *p* < 0.05).

**Table 5:** Effect of dietary histamine on the intestinal antioxidant ability of striped catfish.

Items	H0	H15	H30	H60	H120	H240	H480
T-AOC (U/mg prot)	0.80 ± 0.10 ^b^	0.76 ± 0.02 ^b^	0.77 ± 0.03 ^b^	0.78 ± 0.04 ^b^	0.60 ± 0.06 ^a^	0.58 ± 0.03 ^a^	0.53 ± 0.04 ^a^
POD (U/g prot)	54.22 ± 13.01 ^b^	50.69 ± 10.81 ^b^	45.06 ± 9.33 ^ab^	44.94 ± 7.64 ^ab^	41.12 ± 9.17 ^ab^	39.78 ± 5.97 ^ab^	32.84 ± 9.80 ^a^
CAT (U/g prot)	12.28 ± 1.19 ^b^	10.44 ± 0.36 ^ab^	9.62 ± 0.40 ^a^	9.77 ± 0.06 ^a^	9.56 ± 0.46 ^a^	8.32 ± 1.24 ^a^	8.05 ± 1.20 ^a^
SOD (U/g prot)	33.17 ± 1.75 ^b^	28.74 ± 2.13 ^ab^	31.29 ± 2.27 ^ab^	26.52 ± 5.91 ^ab^	26.12 ± 2.28 ^ab^	23.45 ± 4.24 ^ab^	18.84 ± 1.84 ^a^
GPX (U/g prot)	29.17 ± 2.44 ^c^	27.67 ± 5.00 ^bc^	25.73 ± 2.07 ^bc^	24.94 ± 2.47 ^bc^	22.20 ± 1.97 ^ab^	18.31 ± 3.54 ^a^	17.62 ± 6.23 ^a^
GR (U/g prot)	24.79 ± 2.72 ^b^	23.16 ± 5.13 ^b^	22.40 ± 2.59 ^b^	22.32 ± 2.51 ^b^	19.81 ± 3.04 ^b^	13.94 ± 2.23 ^a^	14.07 ± 2.13 ^a^
MDA (nmol/mg prot)	1.13 ± 0.65 ^a^	1.44 ± 0.35 ^a^	1.58 ± 0.32 ^a^	1.71 ± 0.64 ^a^	2.47 ± 0.39 ^ab^	2.57 ± 0.87 ^ab^	3.57 ± 1.40 ^b^

Data in the same row with different superscript letters indicate significant differences between groups (*n* = 3; *p* < 0.05).

**Table 6:** Effect of dietary histamine on intestinal immunity of striped catfish.

Items	H0	H15	H30	H60	H120	H240	H480
LZM (U/g prot)	1.55 ± 0.25	1.44 ± 0.10	1.57 ± 0.12	1.86 ± 0.05	1.91 ± 0.48	2.10 ± 0.25	2.04 ± 0.31
C3 (mg/g prot)	20.37 ± 2.21 ^b^	16.28 ± 4.40 ^ab^	15.14 ± 5.21 ^ab^	14.28 ± 3.93 ^ab^	15.53 ± 2.33 ^ab^	12.66 ± 1.89 ^a^	11.48 ± 1.01 ^a^
C4 (mg/g prot)	77.78 ± 4.90 ^c^	66.44 ± 2.76 ^bc^	54.89 ± 7.44 ^ab^	51.08 ± 1.69 ^ab^	45.95 ± 12.71 ^a^	43.78 ± 4.66 ^a^	39.81 ± 2.79 ^a^
IgM (mg/g prot)	15.21 ± 1.80 ^b^	13.93 ± 1.56 ^ab^	13.99 ± 3.35 ^ab^	10.82 ± 1.85 ^a^	10.13 ± 0.67 ^a^	10.47 ± 1.24 ^a^	9.57 ± 0.04 ^a^
LPS (ng/mg prot)	0.69 ± 0.14 ^a^	0.84 ± 0.13 ^ab^	0.95 ± 0.01 ^bc^	1.18 ± 0.02 ^c^	1.12 ± 0.17 ^c^	1.15 ± 0.05 ^c^	1.11 ± 0.09 ^c^

Data in the same row with different superscript letters indicate significant differences between groups (*n* = 3; *p* < 0.05).

**Table 7:** Effect of dietary histamine on microbiota α-diversity of striped catfish.

Items	H0	H15	H30	H60	H120	H240	H480
Goods coverage	0.99	0.99	0.99	0.99	0.99	0.99	0.99
Shannon	4.69 ± 0.21	4.35 ± 0.19	4.65 ± 0.11	4.29 ± 0.25	4.38 ± 0.15	4.64 ± 0.58	5.28 ± 0.09
Simpson	0.92 ± 0.01	0.88 ± 0.01	0.91 ± 0.03	0.89 ± 0.03	0.89 ± 0.00	0.89 ± 0.06	0.94 ± 0.02
Chao1	462.84 ± 112.71	503.1 ± 51.95	532.33 ± 42.14	511.99 ± 111.15	492.98 ± 19.82	531.99 ± 33.7	472.69 ± 17.32
Ace	467.86 ± 121.96	507.63 ± 54.8	537.84 ± 46.52	507.99 ± 115.19	499.28 ± 22.27	533.86 ± 30.56	465.54 ± 16.79

## Data Availability

Data is contained within the article and in the Supplementary Material.

## Supplementary Materials

The following supporting information can be downloaded at: https://www.mdpi.com/article/10.3390/antiox11112276/s1, Table S1: Intestinal digestive enzyme activities of striped catfish fed with different experimental diets.
