# Supplementary material for: High Dietary Histamine Induces Digestive Tract Oxidative Damage in Juvenile Striped Catfish (Pangasianodon hypophthalmus)
Source: Antioxidants (Basel). 2022 Nov 17;11(11):2276. doi: 10.3390/antiox11112276 (PMC9686954; doi:10.3390/antiox11112276)
Supplement: Supplementary file 1 [file antioxidants-11-02276-s001.zip › antioxidants-1994355-supplementary.pdf]

Supplementary Material

Table S1. Intestinal digestive enzyme activities of striped catfish fed with different experimental diets.

Item	H0	H15	H30	H60	H120	H240	H480
Pepsin (U/mg prot)	27.15 ± 3.49	26.52 ± 5.43	26.91 ± 2.21	23.5 ± 5.57	24.10 ± 1.97	20.08 ± 2.38	21.77 ± 2.60
Trypsin (U/mg prot)	501.58 ± 1.02^{b}	499.64 ± 21.42^{b}	393.06 ± 47.09^{a}	392.05 ± 12.03^{a}	337.86 ± 18.78^{a}	378.41 ± 38.57^{a}	388.75 ± 24.97^{a}
Lipase (U/g prot)	123.28 ± 13.41^{b}	113.86 ± 19.42^{ab}	116.93 ± 1.58^{ab}	99.71 ± 7.16^{ab}	101.4 ± 13.07^{ab}	99.06 ± 11.68^{ab}	87.75 ± 3.62^{a}
Amylase (U/mg prot)	0.17 ± 0.03	0.17 ± 0.04	0.14 ± 0.02	0.13 ± 0.01	0.13 ± 0.01	0.12 ± 0.01	0.14 ± 0.01
Maltase U/mg prot	233.21 ± 18.27^{c}	214.27 ± 36.30^{c}	203.94 ± 5.59^{bc}	199.47 ± 10.22^{bc}	192.19 ± 8.27^{bc}	137.28 ± 21.62^{ab}	107.94 ± 4.39^{a}

Data in the same row with different superscript letters indicate significant differences between groups ($n = 3$; $p < 0.05$).
